# Ten Years toward Equity: Preliminary Results from a Follow-Up Case Study of Academic Computing Culture

**DOI:** 10.3389/fpsyg.2017.00816

**Published:** 2017-05-19

**Authors:** Tanya L. Crenshaw, Erin W. Chambers, Cinda Heeren, Heather E. Metcalf

**Affiliations:** ^1^Cascade EnergyPortland, OR, United States; ^2^Computer Science, St. Louis UniversitySt. Louis, MO, United States; ^3^Computer Science, Thomas Siebel Center for Computer Science, University of Illinois at Urbana-ChampaignUrbana, IL, United States; ^4^Association for Women in ScienceWashington, DC, United States

**Keywords:** academia, computing, cultural change, gender, policy design, recruitment, retention, STEM

## Abstract

Just over 10 years ago, we conducted a culture study of the Computer Science Department at the flagship University of Illinois at Urbana-Champaign, one of the top five computing departments in the country. The study found that while the department placed an emphasis on research, it did so in a way that, in conjunction with a lack of communication and transparency, devalued teaching and mentoring, and negatively impacted the professional development, education, and sense of belonging of the students. As one part of a multi-phase case study spanning over a decade, this manuscript presents preliminary findings from our latest work at the university. We detail early comparisons between data gathered at the Department of Computer Science at the University of Illinois at Urbana-Champaign in 2005 and our most recent pilot case study, a follow-up research project completed in 2016. Though we have not yet completed the full data collection, we find it worthwhile to reflect on the pilot case study data we have collected thus far. Our data reveals improvements in the perceptions of undergraduate teaching quality and undergraduate peer mentoring networks. However, we also found evidence of continuing feelings of isolation, incidents of bias, policy opacity, and uneven policy implementation that are areas of concern, particularly with respect to historically underrepresented groups. We discuss these preliminary follow-up findings, offer research and methodological reflections, and share next steps for applied research that aims to create positive cultural change in computing.

## 1. Introduction

Over the past 50 years, researchers, policy makers, educators, and employers have invested much toward the recruitment and retention of women and people from historically underrepresented minority backgrounds in computing. While some scientific fields, such as the life sciences, have seen great improvement in degree participation by women, computer science and engineering have experienced declining occupational and degree participation by women and people of color, with enrollment and employment trends by these groups in 2013 nearing where they were in the 1960s (Hill et al., 2010).

This gap is particularly problematic with respect to the shift in the college-going population in the past 50 years (Barr, 2014). The number of bachelor's degrees awarded annually has more than tripled since the 1960s. The proportion of women earning bachelor's degrees has grown from 43 to 57% in 2013; in 2013, 50.3% women earned 50.3% of science and engineering bachelor's degrees (National Science Board, 2016). Yet, that same year, women earned only 18% of bachelor's in Computer Science. In looking at all of the bachelor's degrees earned by gender, in the 1960s men were earning Computer Science degrees at three times the rate of women, while by 2012, men were earning Computer Science degrees at six times the rate of women.

Looking beyond undergraduate degree attainment, other work shows gaps in the workforce. Proportions vary broadly by field, but the computer and information science, and engineering workforces see the smallest proportions of women at 24 and 15%, respectively (National Science Board, 2016). In 2014, Google publicly disclosed that 17% of its tech workers were women, 3% were Hispanic and 2% were black (Huddleston, 2014). That same year, Facebook, LinkedIn, and Yahoo also publicly released employee diversity reports, with similar demographics. On the academic side, in 2010, only 4% of full-time Computer Science faculty were members of underrepresented minority groups (National Science Board, 2014). In 2013, their numbers had risen to 6% (National Science Board, 2016).

Much research explains such gaps by pointing to cultural barriers and biases faced by women and people from underrepresented minority backgrounds. These barriers and biases influence sense of scientific identity, self-efficacy, and fit (Rosser, 2004; Moss-Racusin et al., 2012; Corbett and Hill, 2015). They are also largely responsible for the stratification and inequities related to a complex landscape of professional experiences: Hiring, space and resource allocation, salary and compensation package composition, evaluation, recognition and awards, research grant funding, promotion, tenure, access to key professional networks and mentors, movement into leadership roles, access to funding and knowledge resources necessary for scientific commercialization, and more (Heilman and Okimoto, 2007; Bilimoria et al., 2008; Isaac et al., 2009; Ely and Rhode, 2010; Hill et al., 2010; Ely et al., 2011; Metcalf, 2011; Moss-Racusin et al., 2012; Lincoln et al., 2012; Blume-Kohout, 2014).

Research and practice have focused primarily on the recruitment side of the picture, aiming to draw more women and people of color into STEM by “fixing” their interests, self-confidence, and self-efficacy and providing them with tools they can use to survive within the existing culture (Metcalf, 2011, 2014; Fouad et al., 2012; Knipfer et al., 2017). While this approach can be immensely useful as a support system, it does not address root systemic causes necessitating survival mechanisms in the first place. Survival mechanisms alone cannot and have not resolved the structural and systemic issues pervasive within STEM educational and work spaces. This study joins in the research efforts dedicated to understanding and addressing the cultural and experiential issues impacting the retention of women and people of color in STEM, particularly in computing disciplines where retention issues are growing.

This year, universities around the country, including the University of Illinois at Urbana-Champaign, are reporting record-breaking numbers of freshman women entering Computer Science programs (Hustad, 2016; RHIT, 2016; Williams, 2016). We are excited to see this influx of women due to recruiting and outreach efforts. But we and the department at Illinois want to make sure that the underlying cultural barriers and biases are addressed in ways that facilitate the success of the students and faculty within the department rather than seeing a mass exodus down the road. Even if these women choose to stay in their programs and complete their degrees, there is still much work to do in improving the educational and professional opportunities and experiences of underrepresented groups.

To that end, we report on our Spring 2016 pilot results from a follow-up case study of the Computer Science culture at the University of Illinois at Urbana-Champaign, a research-intensive Computer Science department. Though we have not yet completed our full data collection and cannot generalize our results, we find it worthwhile to reflect on the pilot data we have collected thus far. Pilot data reveal improvements in the perceptions of undergraduate teaching quality and undergraduate peer mentoring networks. However, we also found evidence of continuing feelings of isolation, incidents of bias, policy opacity, and uneven policy implementation that are areas of concern, particularly with respect to historically underrepresented groups.

## 2. Study history

To provide some background on the history of our study, we discuss our original study and the events that led up to our Spring 2016 pilot study.

**Figure 1:**
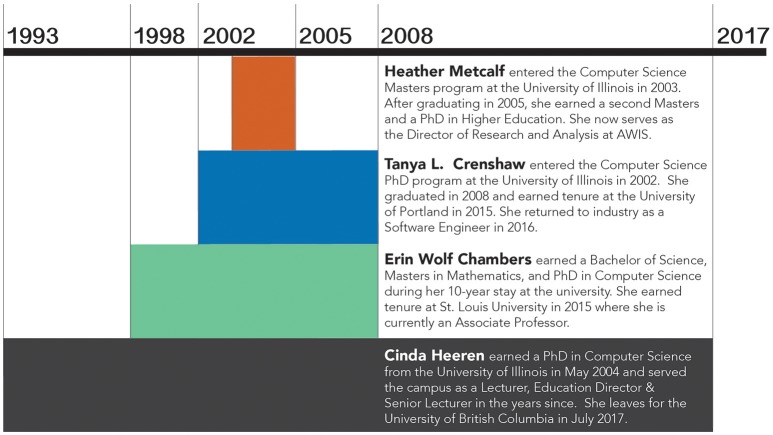
**Altogether, the co-authors have spent 42 years at the University of Illinois at all levels of inquiry: undergraduate, graduate, and faculty**.

Altogether, we have spent a total of 42 years at the University of Illinois' Department of Computer Science at multiple levels: undergraduate, graduate, and faculty. As summarized in Figure 1, we met in 2003, the start of a brief 2-year window during which we were all students in the Department of Computer Science.

In 2005, Heather Metcalf graduated from Illinois and began pursuing her master's degree in Gender and Women's Studies at the University of Arizona. In part, her motivations for seeking the degree was to better and more systematically understand and address a number of problematic gendered and cultural experiences she had had and witnessed while obtaining her first master's in Computer Science at Illinois.

At the time, Computer Science was experiencing a downward trend in enrollment at the national and university levels (Vegso, 2005). Cinda Heeren, working on completing her Ph.D. in Computer Science, was piecing together funding from a variety of positions such as *Visiting Assistant Director of Diversity Programs* and *Visiting Lecturer*. Third-year Ph.D. students Erin Wolf Chambers and Tanya L. Crenshaw were experiencing a sense of unease in the department that aligned with many of the Metcalf's own experiences at Illinois. The department held a disciplined focus on research excellence, but the focus had evolved into a culture of exclusivity that seemed to be having a particularly detrimental effect on women and students of color in the department. Talented students were not feeling successful and were leaving their programs of study.

**Figure 2:**
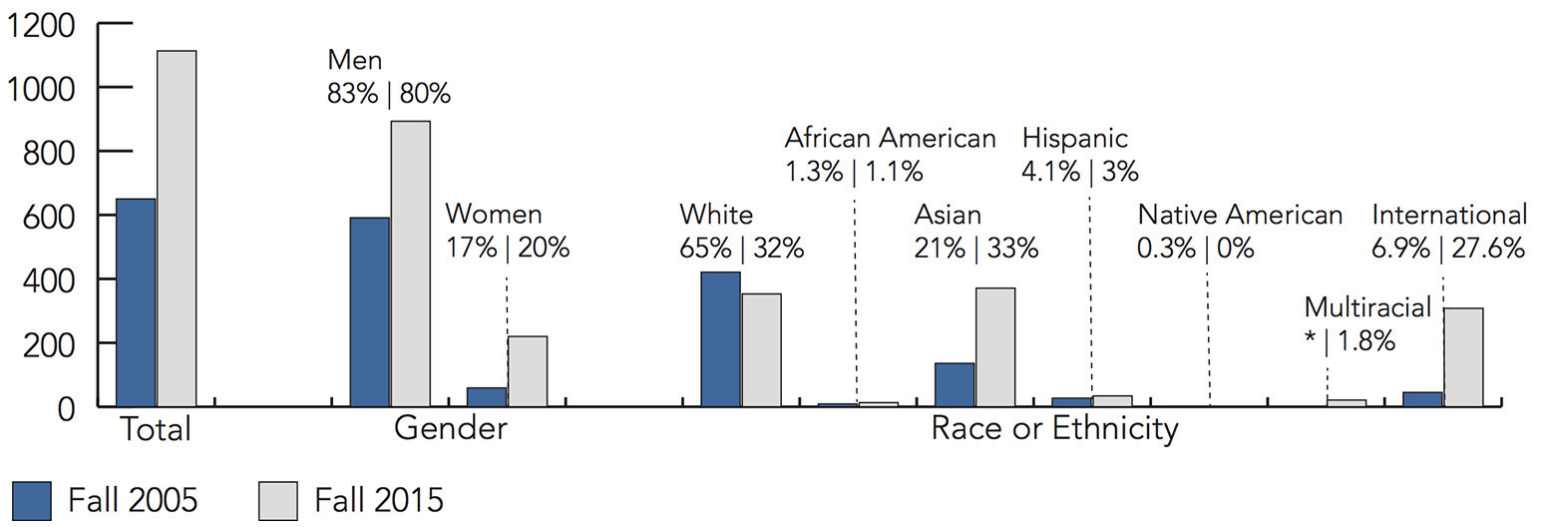
**A comparison of the undergraduate student demographics in Fall 2005 and Fall 2015**. In 2005, there were 650 undergraduate students. The population almost doubled by 2015, with 1113 students. Note that “Multiracial” was not a racial category measured by the department in 2005.

**Figure 3:**
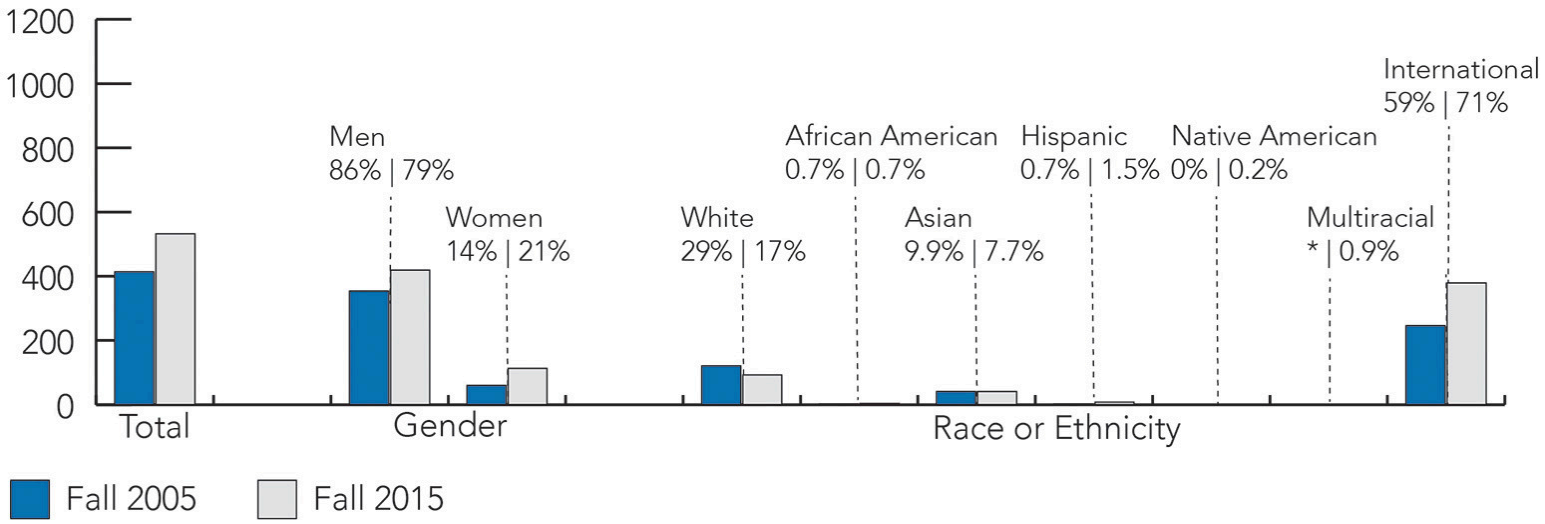
**A comparison of the graduate student demographics in Fall 2005 and Fall 2015**. In 2005, there were 414 undergraduate students. By 2015, the population was 532 students, with 21% women and over 70% international students. Note that “Multiracial” was not a racial category measured by the department in 2005.

**Figure 4:**
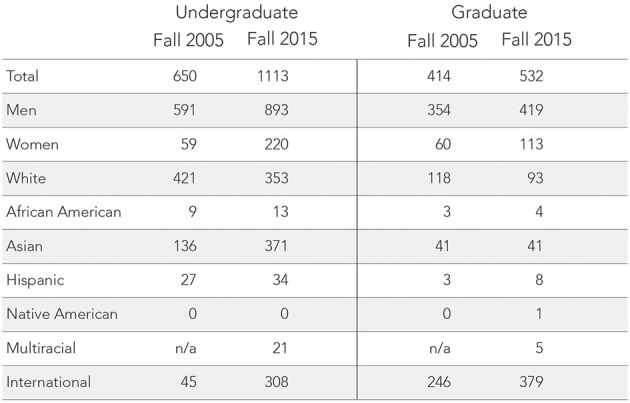
**A tabular summary of the column data visualized in Figures 2, 3**. From 2005 to 2015, the undergraduate population almost doubled while the graduate population saw an increase of 28%. Note that “Multiracial” was not a racial category measured by the department in 2005.

Compounding this sense of unease was the demographic landscape of the department. As shown in Figures 2–**4**. in Fall 2005, the department's undergraduate population comprised 650 undergraduates and 414 graduates. Men comprised close to 90% of both the undergraduate and graduate populations.

After seeing many brilliant friends and colleagues leave the department and the field, we felt action was necessary. Across two time zones, we self-organized into a research team and sought to understand the departmental culture beyond our own individual experiences. From January to July 2006, we conducted a two-phase study to evaluate undergraduate and graduate student attitudes on areas contributing to enrollment and persistence: recruitment, retention, and preparation. We began the study by interviewing eleven participants, deliberately picked for their collective breadth of experience and demographic characteristics. These hour-long interviews uncovered qualitative trends and helped to refine the survey questionnaire. In the second phase, we utilized online questionnaires that collected quantitative and qualitative data to survey a total of 119 students comprising 61 undergraduate students, and 58 graduate students. Overall, the survey participants were 17% women and 83% men, at a time when the department itself was 14% women and 86% men.

In our 2006 study, we found that while the department placed an emphasis on research, it did so in a way that devalued teaching and mentoring, and negatively impacted the professional development, education, and sense of belonging of the students. We found a lack of communication and transparency throughout the department fueled these frustrations. While these negative impacts were expressed to a slightly greater degree by women in the department, they were pervasive across all demographic categories.

By the end of our work, we presented the department with a collection of policy and practical recommendations. Published in a white paper to the department (Crenshaw et al., 2007), a 2008 article (Crenshaw et al., 2008) and in Metcalf's second master's thesis (Metcalf, 2007), these recommendations encouraged more interactions among community members, greater flexibility in programs of study, and more opportunities for quality outreach, teaching, and mentoring that can also assist in the department's research orientation and goals. The eight recommendations were:

1. Provide more comprehensive information to prospective graduate students.
2. Facilitate more opportunities for outreach.
3. Facilitate more interaction between students and faculty.
4. Improve quality of teaching.
5. Provide more flexibility in core requirements.
6. Increase early research opportunities.
7. Create multiple and diverse mentoring opportunities.
8. Provide an adequate family leave policy.

In the years since the original 2006 study, through the bold involvement and advocacy from several key faculty members, the department has revised its curricula, advising policies and practices, and outreach efforts. Quoting a letter of support from the Computer Science Department Head (Rutenbar, unpublished), the specific changes informed by our study included:

1. A smaller and more flexible undergraduate core curriculum.
2. A more flexible graduate core curriculum, which again attracts students with more diverse backgrounds and interests, and which allows Ph.D. students to start research more quickly.
3. Annual progress reviews for all Ph.D. students, which have improved communication between students, advisors, and other faculty mentors.
4. Changes in graduate admissions procedures which broaden the set of potential advisors for all incoming students.
5. A formal teaching requirement for all Ph.D. students.
6. Significant improvements in undergraduate advising policies, including an assigned Faculty Mentor to every undergraduate as well as two full time student-facing academic professionals.

While the department instituted a number of changes, many were met with resistance. During a presentation of the findings, several faculty members expressed disbelief even in the face of statistically significant evidence. We experienced a particularly poignant moment during one presentation to the faculty. While discussing a participant's quote on a lack of mentors, one faculty member said, “*Your work is very interesting, but this kind of thing doesn't happen in my research group.”* We had interviewed two of his students, one of whom said the exact quote to which he objected. We knew that “*kind of thing”* happened in his group, and that his own students were lamenting a lack of research mentors. We report only one of our own experiences here, but this pattern of resistance despite empirical evidence is seen in the research on bias. Both men and women have relative reluctance, particularly those who are faculty in STEM departments, to accept evidence of gender biases in their field (Uhlmann and Cohen, 2005, 2007; Castilla and Benard, 2010; Handley et al., 2015).

In the changes that have been made, it is unclear how deeply or how meaningfully the culture has improved as a result. In addition, new challenges have arisen; the CS department has grown considerably in size. In response, in 2015, the department's Associate Department Head reached out to our team to conduct a follow-up study to gain a renewed sense of the current state of the departmental culture. Cinda Heeren, now one of the first official teaching faculty in the department and one of the first to be subject to the professionalization of the instructional track, joined our original team to contribute her longitudinal, pedagogical, and advocacy expertise to the design and implementation of this study.

**Figure 5:**
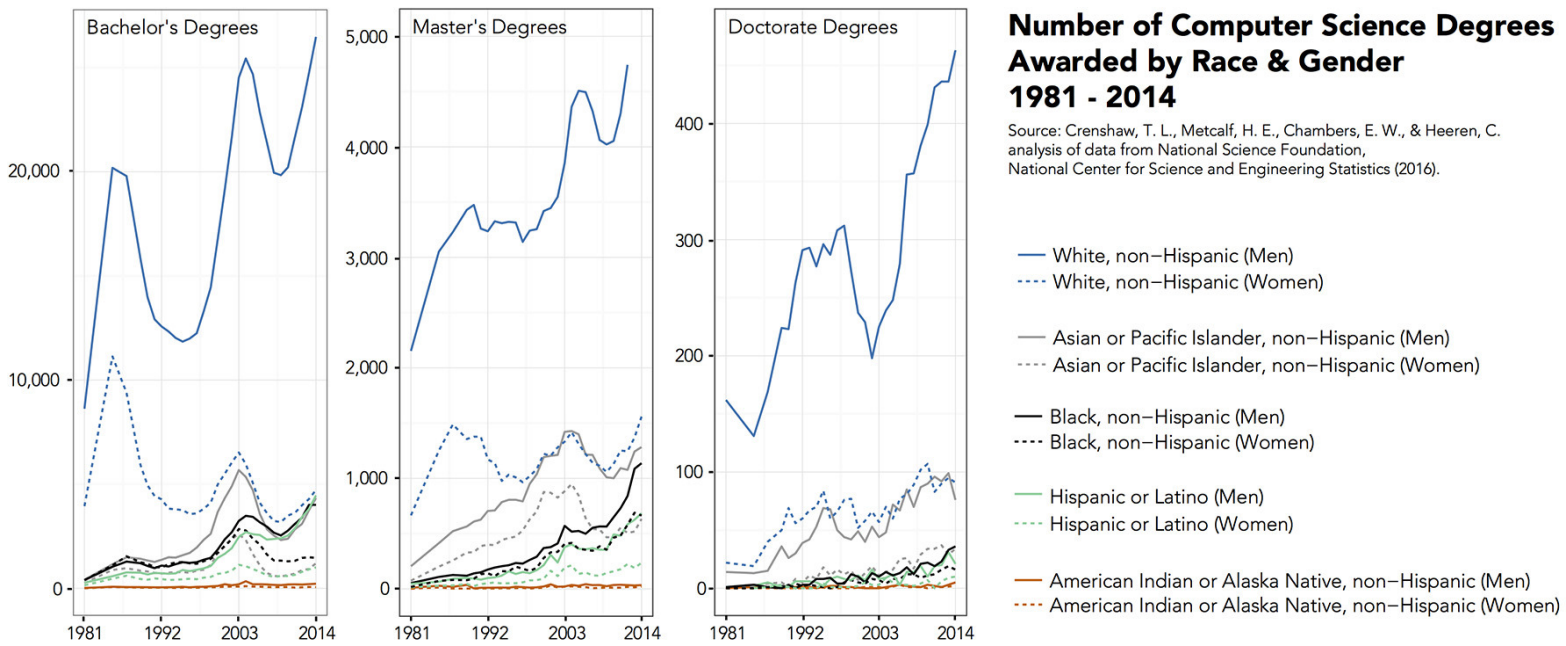
**Nationally, the number of Computer Science degrees awarded by race and gender from 1981 to 2014**. Data source: National Science Foundation, National Center for Science and Engineering Statistics (2016).

Over 10 years later, the demographics nationally and departmentally have shifted. As shown in **Figure 5**, Computer Science is seeing increasing enrollments. Universities around the country, including Illinois, are reporting record-breaking numbers of freshman women entering Computer Science programs (Hustad, 2016; RHIT, 2016; Williams, 2016). Between Fall 2005 and Fall 2015, the Department of Computer Science undergraduate population almost doubled at 1113 students (893 men, 220 women, 353 white, 13 African American, 371 Asian, 34 Hispanic, 21 Multiracial, 308 international, 13 “unknown,” and 474 state residents). There were 532 graduate students (119 MCS students, 103 MS students, 310 doctoral students) with demographic descriptives summarized in Figures 3, 4. This time, women comprised roughly 20% of the student population which is similar to national student trends seen in Figure 5. Students from under-represented minority backgrounds represented 6.2% of the undergraduate population (8.6% among U.S. citizens) and 3.4% of the graduate population (11.8% among U.S. citizens), which is about one quarter of the level of representation in the national trends.

## 3. Methodology

In our current iteration of this work, we are taking a critical mixed-methods approach to a case study of the computer science departmental culture (Creswell, 2008) at the University of Illinois at Urbana-Champaign. Our approach pairs survey data with in-depth, semi-structured follow-up interviews of current undergraduate students, graduate students, and faculty about their experiences with various aspects of the department's culture. To develop the survey instrument and interview protocols for this inquiry, we began with the original 2005 instruments, updating them to reflect any revised or new university programs and policies. Updates to the instruments also incorporated findings from the research on recruitment, retention, and persistence into the workforce that have been released since 2005. In addition, we created new survey and interview protocols for use with departmental faculty to incorporate their perspectives and experiences for a more holistic view of the culture. This was impossible while we were all graduate students in the department, but we believe it is a piece of the picture that was missing in 2006.

Before distributing the surveys and conducting their subsequent follow-up interviews throughout the department, in Spring 2016, we piloted our revised instruments with a deliberate sub-sample of undergraduate students, graduate students, and faculty from the department^1^.

In our participant solicitation for the pilot study, we selected participants to reflect a breadth of experiences, from first-year undergraduates to tenured faculty, and demographic characteristics. We made clear our own connections to the university. Moreover, due to Heeren's extensive student network and pre-existing relationships with the students and faculty, our recruitment and consent documents made it clear that she would not be present during the interviews, nor would she have access to the resulting transcripts or identifying data.

Eleven participants (four undergraduate students, five graduate students, and two faculty) were sampled, using an e-mail solicitation sent by Heeren to 18 members of the department. Participants were given a short background of the study, including a message that participation in the study was entirely voluntary. Individuals agreed to participate in the study by scheduling an appointment with the three interviewers: Chambers, Crenshaw and Metcalf. We spent 1 h to 90 min with each participant; we administered a paper version of our pilot survey instrument and conducted semi-structured follow-up interviews based on the participant's survey answers.

This paper reports on the pilot study results, focusing on preliminary qualitative findings from the undergraduate and graduate student populations. While we have developed our survey and conducted pilot interviews with two faculty, here we focus on our qualitative student data for the purposes of preliminary comparison between the 2005 study and the current departmental patterns. We will continue to pilot the faculty instruments and will report on these findings in future work.

For the undergraduates, the survey instrument covered topics related to their pathway to the department, their courses, the quality of teaching they experienced, their academic advising needs and experiences, their mentoring needs and experiences, sources of support, policy awareness, their sense of fitting in and feeling successful in the department, extracurricular activities, departmental values, future career goals, and demographic information. Survey questions included open-ended response options for demographic questions and at the end of each topic area so that participants could share more freely about their experiences with that topic in the department. For the graduate students, the survey instrument covered the same topics in addition to graduate program requirements, choosing an area of research, and, for doctoral students, the Ph.D. qualification exam.

For the analysis of our qualitative data, we conducted inductive thematic content coding (Braun and Clarke, 2006) and critical discourse analysis (CDA) (Ayers, 2005; Fairclough et al., 2011) of over 94,000 words of interview transcripts. As has been done in past qualitative work utilizing inductive thematic content analysis (McClelland and Holland, 2015; Moss-Racusin et al., 2015), the expansive body of research on culture in computer science, particularly related to academia, has been used to guide the analysis such that specific thematic categories arose organically from the data. Using the cloud-based software app Dedoose, two members of the research team were responsible for independently coding the data, beginning with a pilot test on a sub-sample of the data. Coding categories and themes were iteratively cross-checked for inter-coder reliability and to resolve coding discrepancies. Our analysis has helped us to refine the survey we will administer to the department in the Spring 2017 semester.

The critical approach that we take is one that emphasizes the importance of taking ownership of our own subjectivities and how these influence our research inquiry. Critical researchers, qualitative and quantitative alike, advocate that scientific research include a “systematic examination of powerful background beliefs” (Harding, 1991) that shape the work. These scholars charge researchers to engage in critical self-reflexivity, claiming responsibility for their positionalities by acknowledging them throughout their work (Haraway, 1991; Harding, 2006; Baez, 2007). To that end, our methodological approach also includes Section 5 of this paper, a reflection on our positionalities relative to the study and the experiences shared by our participants.

## 4. 2016 Pilot study results

This paper details the preliminary comparisons based on our longitudinal work. Though we have not yet completed our data collection and cannot generalize our results, we find it worthwhile to reflect on the pilot data we have collected thus far as they illustrate compelling qualitative patterns thus far. Our analysis revealed three major themes:

- Improvements in the perceptions of undergraduate teaching quality.
- Improvements in undergraduate peer mentoring networks coupled with continuing feelings of isolation at both the undergraduate and the graduate level.
- Incidents of bias exacerbated by policy opacity and uneven policy implementation, all areas of concern, particularly with respect to historically underrepresented groups.

We discuss these themes juxtaposed with the recommendations we made in 2006. Though our preliminary results do not align with all eight recommendations, these three are the most relevant:

- **Facilitate more interaction between students and faculty**. Multiple student participants described an absence of meaningful interactions with faculty. They expressed interest in increased interactions in advising and mentoring, in graduate classes, and with respect to activities, like the qualification exam. This section covers the qualification exam, where positive faculty interactions should occur and have great impacts on retention and success. Some participants reported direct mentorship during their qualification exam. Yet, others expressed a lack or complete absence of feedback on performance and described uneven implementation of recent changes in the qualification exam format.
- **Improve quality of teaching**. Additions of teaching staff and teaching assistant training have lead to positive perceptions of teaching in the 100- and 200-level courses for undergraduates among our participants. Still, our participants described remaining gaps in graduate-level teaching quality and in teaching assistant preparation.
- **Create multiple and diverse mentoring opportunities**. Each of our student participants described challenges in finding effective tenure-track faculty mentoring relationships and sought creative means to develop career and psychosocial support networks for themselves. For our undergraduate participants, departmental and university student organizations remained places where they find mentoring relationships with their peers. However, graduate student participants, as in the original study, expressed feelings of isolation.

We discuss these recommendations and their underlying themes in greater detail in the subsequent sections.

### 4.1. Facilitate more interaction between students and faculty

In 2006, the final question of the survey asked simply, “What do you think could be done to improve the department?” Of graduate participants, 31% cited increasing student-professor interactions. In a department whose student population has grown as much as Illinois', and without new hires keeping pace, it is increasingly difficult for faculty to balance their existing workloads, let alone to make time for additional student engagement. That said, there are moments already in the rhythm of the academic year where greater interactions may be more purposefully had.

One such moment is the Ph.D. qualification exam.

#### 4.1.1. The Ph.D. qualification exam

The Ph.D. qualification exam is the first major milestone for doctoral graduate work in the Computer Science department. According to the department's public website,

“*The purpose of the Ph.D. Qualifying Exam is for students to convince the faculty that they should be considered a Ph.D. candidate. Faculty evaluate whether the student has the knowledge, experience, perspective, and determination to complete the Ph.D. program. In addition, faculty will evaluate the student's presentation and communication skills to ensure a mastery of English sufficient to teach in a U.S. institution can be achieved by the end of the Program*^2^*.”*

Publicly, the department offers that implementation of the exam may vary across research groups. It certainly varies. Comprehensive written tests, oral presentation of assigned research papers, submission of an original manuscript, and unstructured interviews are all examples of how the exam is implemented across groups. For some students, the exam format selected by their research group was effective, efficient, and contributed to their professional development.

An international second-year Ph.D. student described the recently revised format of her qualification exam, “*We need to just write a paper…about our own research.”* She presented her work in a Q&A format to four professors in a committee. She said of the format, “*And I really liked it that it was to write a paper, rather than spending time doing course work that doesn't really help with the research. So I was really happy with the new style.”*

She also described the kind of feedback she received after the exam, “*I got the paper reviews for each of the professors with all of the comments and I have to rewrite my paper. They were expecting things to be there that I didn't explain well. But it's rewriting a paper that I would potentially publish and I got reviews before actually publishing it.”*

For this student, the exam format was an opportunity to receive feedback on her research, writing, and scientific communication skills and helped her strengthen her work. She was being asked to complete a task she would need to do well to complete her Ph.D.: Write research papers. In the process, she interacted with four professors, discussing her own research, 2 years into her degree. Moreover, she received high-quality written feedback in the same form that she'll receive on her future scholarly works, even after graduation. She said, “*The best part was it was about my research so whenever I go back to [the committee's] questions, I feel like there was an improvement or new ideas about my own work, which is the most important thing about research.”*

Other students, however, did not experience quality interactions with faculty through their qualification exams. For example, a white third-year doctoral student described his format as a written exam based on a reading list. He said, “*‘You passed’ was my feedback. Never got to see my exam. Never got any detailed opinion of what things I was good at, what things I should improve on. Which papers I did better at, which ones I didn't do as good at.”* This student expressed a strong desire to have the opportunity to reflect on his strengths and weaknesses and felt that the lack of feedback served as a barrier to being as successful as possible. From his perspective, even though he passed, the exam did not contribute to his professional development nor did it allow for meaningful engagement with his research group advisor or faculty committee members.

An underrepresented doctoral student of color in her third year, who also took a written exam, received a conditional pass. She said, “*The thing is, like they don't tell us how we passed. I just got [word] that I passed on the condition that I have a research paper submitted by the end of next semester. But there's nothing that says you did well on the written part, but you didn't do well on this part. Or you did well on this part on part but you didn't do well on this specific question.”* Like the previous student, she sought a deeper understanding of skills so that she could work to improve upon them and expected that the qualification exam would present her with that opportunity from faculty feedback.

Given that this is the first of only three milestones in the Computer Science Ph.D., it is concerning that students are not receiving quality feedback. It is a missed opportunity for greater interactions between students and faculty in the department that have a direct impact on the students' success. The qualification exam is a moment that can provide students with quality professional feedback and direct mentoring that would aid in their development.

An area of even greater concern is how implementation of the exam can differ for students from different demographics even within the same research group. We interviewed two graduate students from the same research group comprised of about 10 tenure-track faculty members. From a policy transparency, implementation, and equity perspective, interviews with these two students stood out.

In both cases, the students were offered a two-part exam. In the first part, the group's faculty offered a standard reading list 3 months before a closed-book written exam was administered. The purpose of the written exam was to assess student knowledge of the research area's fundamental topics.

The second part of the exam was described in this way by a white doctoral student,

“*There was a verbal part, but basically, my understanding of what that was for was purely just to evaluate your English speaking skills. It was like, meet with a faculty member and talk about your research for like 10 min.”*

When asked how many meetings, the student replied, that he was supposed to have three independent meetings, and that, “*I think I ended up having two instead of three. Because one person was just not available*.

The same exam was described by an underrepresented woman of color,

“*Part of my qualifying exam is taking the written exam and then basically going to each and every one of the [group's] faculty and having a 10–15 min interview which covers absolutely nothing.”*

This student also described bias and assumptions in her day-to-day interactions with faculty and students, particularly around language:

“*I'm American and I'm also [race]. I know how to speak English very, very well. It's my first language. I also know how to speak [another language] very, very well. I'm a bilingual. But they never dare to ask. And I feel like its ok. Don't be too afraid to ask, you know. As long as like, you're asking honestly and not trying to, you know …I don't know. There is an honest way of asking a question. And then there is a way where you're like just looking for a fight.”*

Both students in this research group separately described unwritten evaluation criteria for the verbal part of the exam that made them both feel uncomfortable because of the ways these criteria target international students. Both also expressed the sense that this portion of the exam was providing no feedback and serving no larger purpose in the evaluation of their research skills.

### 4.2. Improve quality of teaching

With respect to teaching, great changes have taken place as a result of the policy changes in the past 10 years. In our original study, 45% of undergraduates did not feel the department valued teaching, and 66% did not attend class because they felt that lectures did not help them learn the material. Ten years ago, over half of the suggestions from undergraduates were related to teaching improvements.

Since 2006, two notable changes have been put in place. First, the department has introduced a professional teaching track position; teaching faculty hired into this track handle the bulk of introductory undergraduate level courses. Second, Ph.D. students are now required to fill a teaching assistant role for one semester. With that, there is now a “Teaching Assistant Preparation” seminar required for beginning Ph.D. students who run labs or teach discussion sections. We discuss these changes in turn.

#### 4.2.1. Professional teaching track

In 2006, a substantive number of student comments on the department were related to teaching. One participant, whose sentiment reflected much of what our sample population reported, said,

“*I would like the university to help teach professors who are great minds/researchers how to teach and interact with students better. I understand that UIUC is a great research school, and I know the importance of this, but sometimes the people who are best at research are terrible teachers.”*

At the time, we offered that one solution could be to adopt the “Assistant Professor of the Practice” position (Fogg, 2004) that were seen at universities like Duke and Carnegie Mellon University. Today, the university offers a variety of teaching faculty positions. As described on a recent job opening at the department's website,

“*Teaching faculty positions are renewable, career-oriented, non-tenure-track positions. Initial appointments are typically at the rank of Instructor, Lecturer, or Teaching Assistant Professor, with the possibility of promotion to the ranks of Senior Instructor, Senior Lecturer, or Teaching Associate Professor and Teaching Professor*^3^*.”*

Within the department, these kinds of teaching faculty are responsible for the CS1 and CS2 introductory programming sequence taken by all undergraduates. Almost all students interviewed in 2016 felt that the department valued teaching, although many noted that it valued teaching only at lower levels, where non-tenure track faculty emphasized teaching as the main part of their job. An underrepresented graduate student of color who works as a teaching assistant for one these courses describes them in this way, “*CS 125 and CS 225, they're kind of like low level, freshman, sophomore, kind of level courses. And these are excellent courses. They're envisioned excellently. They have the best professors, the best lecturers in the whole department. But once you, climb up into 400 and 500 levels just goes (whew sound). You know? And its more of…sometimes I feel like if we're—especially for the 500 level classes. It's not about the teaching, it's more about the showing.”*

Another graduate student, a white man, echoed this sentiment, “*I think the Computer Science department has a split brain. And one half of that brain really values excellent teaching. And the other half of that brain couldn't care less.”* An international graduate student was more precise. When describing the teaching quality in the upper division courses, she said, “*It depends on the faculty. Because I've taken another 500 level course and that professor was excellent.”*

Thus, it seems that the department's investment in undergraduate teaching, particularly at the lower-levels, has helped to shift the perception of undergraduate teaching quality and investment. These investments were not matched at the graduate level, where our 2016 participants continue to express high levels of dissatisfaction in the quality and level of investment in teaching. In addition, this investment does not pay adequate attention to equity issues in terms of who is taking on these roles and how the roles are valued financially, as many of the teaching staff positions at the university seem to offer less job security and lower pay; retention of high-quality teaching faculty may be a challenge on campus.

#### 4.2.2. Teaching assistant preparation

In the original study, the quality of teaching was a much-discussed topic among participants. Only 65% of participants felt that the department valued excellent teaching. Among the undergraduates, more than half of the recommendations on how to improve the department were related to teaching. At the time, one participant said,

“*I would like to see T.A.s with more instruction on how to teach a class. The first course a student takes in Computer Science is the most critical, because it is that course that will make a student decide whether to stay in the department.”*

Teaching assistants are assigned to a variety of courses with many different demands; some positions require administrative work, grading or assisting students during office hours. Other courses require leading a laboratory or discussion section. In 2006, teaching assistants for the department participated in a campus-wide 2-day training course. International teaching assistants received an additional 2 days of training. At the time, we pointed out that the University of California at Berkeley offered its own course, CS301, called *Teaching Techniques for Computer Science* which discusses techniques for effective teaching specific to computer science.

Today, all Ph.D. students in the department are required to fulfill a teaching assistant role for at least one semester. In response to our recommendation, to better prepare their teaching assistants, the department offers a seminar course, CS591 TA, or “*Teaching Assistant Training,”* to prepare its teaching assistants for their service. Student interviews enumerated some of the course's topics, including lesson planning, encouraging student participation, protecting student privacy, cultural sensitivity and Title IX compliance.

Among the three graduate students interviewed who took the course, two perceived it as useful. One student, a white man, was not as engaged. He felt it was a mix of “*common sense,”* topics he did not care about, and some topics of interest unrelated to teaching. He ended his description of his experience with, “*Maybe I'm not the perfect candidate for that class. Or maybe I just didn't receive the instruction well.”*

While there is unevenness in terms of how the students are engaging in the course, there are also discrepancies in how the seminar requirement is enforced. Of the five graduate students interviewed, two, both men, reported they did not participate in the seminar before accepting a teaching role in the department. One international student offered, “*I actually skipped it and I was allowed to skip it because it's offered only in fall and I wasn't a TA in Fall '14 when I joined, I was an audit in Fall '14. But then I chose to be a TA in spring and then that…seminar wasn't offered in spring.”*

The student went on to explain that his course evaluations offered positive student feedback and so he was not made to take the seminar in Fall 2015. He further offered that he might have taken it in Fall 2015, “*but it clashed with one of my courses. And then I couldn't …I mean I thought I shouldn't be dropping the course for TA training.”*

Those students who had taken the course also identified gaps in their preparation for teaching roles. One white third-year Ph.D. student described his career goal as “*tenure-track faculty member”* was frustrated by the lack of teaching preparation and opportunity he had found in the department. He offered, “*Because I don't think you can just grade your advisor's exams and then say “I'm a teacher now.”* An underrepresented student of color and second-year Ph.D. student described “*heartbreaking”* stories she heard from students during her office hours. She wanted to help, “*but I have no idea where to refer them for example, for mental health or counseling. I don't have that information except like if I actually go and search on the internet.”* She felt very unprepared to handle emergency or crisis situations presented by her students and described a lack of awareness of the departmental or campus resources to which she could direct her students in these moments.

Undergraduate experiences echoed such gaps in teaching assistant training for crisis situations. A senior undergraduate student, a white woman, described one such experience. Led by a teaching assistant, the students were instructed to work in groups on an in-class assignment. She said she got behind on the problem, and started working on it independent of her group. As the teaching assistant circled the room, she saw the student's work and said, “*‘good job, you figured this one out, don't tell these guys because they need to work it out for themselves too.”’* The student went on to say, “*And this kid in my group got so mad that I figured out this problem, he literally stood up, threw his chair back and was like, “you're just a freaking girl, you must have cheated on this thing, there's no way…”*

Nobody did a thing. Met with a roomful of silent bystanders to the violence, the yelling, the insults, the chair-throwing, she said, “*I just left the class crying.”*

Many of the students in our pilot interviews described situations in their courses, online discussion groups, student organizations, and research groups where they witnessed or directly experienced hostile behavior, harassment, and bias and felt unsure about how to handle the situations as teachers, students, or peers.

For example when asked about whether she had witnessed inappropriate behavior on campus, an international graduate student spoke generically about seeing such behavior from both students and faculty in the context of coursework and social interactions. We asked her, “*How do you handle that when it happens?”* She offered that sometimes it can be useful to call out the behavior, and that she had once seen a person do so effectively. She said, “*but that person wasn't from computer science. That was funny to see…they were from physics.”*

More often than not, participants explained that these situations are met with awkward silence. Several of our participants described how the culmination of such experiences, particularly when they have been the target of these words and behaviors, has had a deflating effect. For example, one white undergraduate student explained that she has had to learn to tolerate these behaviors:

“*I don't feel a sense of…of hope that like, oh you know, just push through and it's going to get better, once you get into industry — no. It's just how it is and you have to get used to it or get out of it. It's not really something that's going to be fixed within the next few years.”*

She went on to explain that these experiences profoundly changed her formerly exuberant approach to outreach:

“*Obviously if young women have aspirations to be in this field I want them to succeed and I want to help them achieve their goals. But for the young women who are not sure, I'm not going to push them to do something that they're not sure about…you have to be 150 percent sure that this is what you want or you're not going to succeed and then you've just wasted time…I came into this department putting in a lot of effort into…getting women excited about this and we're going to make a change by just showing them how exciting this all is. And to some degree I think that can work. But, on the other hand…[being here is] not a healthy thing.”*

### 4.3. Create multiple and diverse mentoring opportunities

Our 2006 study uncovered a student appetite for multiple kinds of mentoring relationships, but a lack of opportunity to find mentors. At the time, 18% of undergraduate and 53% of graduate students reported having a mentor, with many participants wishing for some kind of mentor but feeling unsure as to how to find one.

There are multiple ways that a department may offer mentoring relationships to its students. One is through undergraduate advising. However, in 2006, only 2 undergraduate participants, or 3%, said that their advisor also served as a mentor to them. At the graduate level, thesis advisors may also serve a mentoring role, but of the 51 graduate participants who reported having an advisor in 2006, roughly half reported that their advisor was their mentor.

In a university context, tenure-track faculty are often counted upon to serve in a mentoring capacity. To that end, the department has instituted a new role, “*Faculty Mentor,”* to support undergraduate advising. Yet, in a department as large as Computer Science at Illinois, we have great empathy for how spread-thin faculty can feel. The undergraduate population has doubled in 10 years, with few new positions added due to state budget constraints.

With such challenges, it becomes even more important to provide multiple and diverse mentoring opportunities. The student population at any department can serve as a rich network of peer mentoring; senior undergraduates can mentor first-year students and Ph.D. students can mentor those in the Master's program. Students of all levels can learn from each other. Our preliminary results provide evidence of how students do mentor each other as well as the challenges that students feel in reaching out.

We discuss our preliminary results regarding Faculty Mentors and peer mentoring in turn.

#### 4.3.1. Faculty mentors

Since the original 2006 study, the department has instituted a new role, Faculty Mentor. The departmental website on undergraduate advising offers that,

*The Computer Science Department has a three-tiered approach to advising: the Office of Undergraduate Programs, faculty mentors, and peer advisors*^4^ …*.All students are assigned a faculty mentor, with whom they must meet at least once each academic year, typically before April. The department enforces this requirement with a registration hold*^5^.

In the survey for undergraduate students, we asked, “*Why do you meet with your Faculty Mentor?”* Focused on the required nature of the relationship, all four undergraduates wrote similar responses. One wrote, “*Because it's required.”* Another wrote, “*I seldom do; I only do when required. [The faculty mentor is] more focused on checkboxes than individualized advice* + *connections.”*

In undergraduate interviews, there were multiple kinds of Faculty Mentor experiences, from helpful to contractual to discouraging. A junior undergraduate who identified as Asian American said, “*I actually just visited my faculty mentor a few days ago. And it was actually a little more helpful than I thought.”* He described that the Faculty Mentor was meeting with multiple students in a group setting. He said, “*I was like ‘yea, which courses should I take?’ and then in addition to that, he explained what they were about. And…other students were there and they also gave me some feedback…And a lot of them were seniors and upper classmen so, they were pretty good.”*

A junior international undergraduate student said her Faculty Mentor was “*really helpful.”* She recalled a time that she expressed anxiety about her career opportunities and that “*My mentor talked about…that I don't need to feel so pressured to do everything technical. She says 'Just do what you want. During the summer, you don't have to do an internship.' She like calmed me down a bit and assured me.”*

She lamented that her friends did not seem to have the same kind of helpfulness in their Faculty Mentors. “*[Their] Faculty Mentors don't email them until the very end and then like now time's running out. So they just do like a quick 10 min thing and it”s something to get over with. I know a lot of people where it's like that.”* Another undergraduate, who identified as white, echoed the “checkbox nature” of his Faculty Mentor relationship. “*It was a little bit of advice and a lot of more or less review, to kind of say “you”re doing fine.”*

A senior undergraduate student, who identified as white, said that her Faculty Mentor told her that she was “*not cut out for this department.”* In response to an average grade in a course, the Faculty Mentor encouraged her to stop any extra curricular activities. The student said the the meeting was, “*Completely unhelpful.”* She reflected that if she hadn't had her other chosen mentors, if she had been a less resilient student, she may have dropped out of the program because of the interaction.

When asked if she looked into getting a different mentor assigned, she reported that she did not. Furthermore, “*I never knew if I could or not.”*

#### 4.3.2. Peer mentoring

In 2006, there were a handful of departmental organizations providing academic, research, social support and outreach opportunities for students. The organizations that still exist today are:

- CSGSO: The Computer Science Graduate Student Organization. Its main offering is a Friday social hour for graduate students.
- ACM: Association for Computing Machinery student chapter. It is open to both undergraduate and graduate students, though its membership is largely undergraduates. A large physical space in the computer science building is reserved for this student club and is often open as a student lounge and homework space. In addition, the ACM students organize an annual tech conference, *Reflections Projections*. The October 2016 conference had 1,800 registrants.
- WCS: Women in Computer Science. This organization works for recruitment and retention of women at all levels of computer science. WCS hosts meetings and social events for both undergraduate and graduate students. Their largest offering is the ChicTech program designed to inspire girls to consider careers in computing. The November 2016 ChicTech retreat saw 75 high schoolers staying overnight on campus to get a feel for the different opportunities in computer science.

While not created for the purpose of peer mentoring, we find that these departmental organizations provide students with opportunities to meet other students in the department; such interactions allow students to create social networks that can be leveraged for both psychosocial support and professional development.

In 2006, though graduate students seemed largely unhappy with the activities organized by CSGSO, undergraduates enjoyed the organizations in which they participated. Ten years later, the graduate students still wished for more interactions. Undergraduates have a greater number of organizations in which they participate and flourish, but some had not yet found a group with which they “*clicked.”*

In the 2016 interviews, a white senior undergraduate listed the student clubs in which she was involved, “*Hack Illinois, Women in Computer Science, ACM.”* She had even started a new student club specializing in a certain kind of software development. She attributed her club with being, “*a major part, if not the only reason I stayed in this field.”* With all her extra curricular activities, she found that, “*I have friends who are kind of coming to me with career questions and want like, recommendations on those kinds of things.”*

A multiracial senior undergraduate who had transferred into the department from another on campus really liked how many side projects the ACM students had. He said, “*People are really enthusiastic and really into the projects and I love that kind of a culture because it drives me.”*

Of the five graduate students interviewed in 2016, only one mentioned CSGSO. An international Master's student said, “*I mean, I have really limited number of interactions with graduate students which is something that should be happening. So CSGSO tries to keep its happy hour…I try my best to be there, but there's only like five or six people there. Generally it's like the pizza comes at 4:45, people come in at 4:40, maybe 4:42 or something. They grab the pizza and they just leave.”*

This same student lamented his lack of opportunities to interact with other students given that he was in the Master's program. He offered, “*As far as the department is considered, I think there's more social events for Ph.D.'s students than Master's students. Because my roommate is a Ph.D. student…and he has a lot of social events at the department which are hosted by the department.”* He imagined a gathering in which he had the opportunity to meet other kinds of graduate students. “*Let's say I'm a second year Master's and I know someone who's doing a Ph.D., who's in their fourth year, I can look up to them as a mentor or something.”*

Other graduate students echoed this sentiment of “*a limited number of interactions.”* An international graduate student who had spent some time in the Math Department before coming the Computer Science compared how the two department provided “shared spaces to graduate students.” As a smaller department, she said of math, “*The first year, the department puts all the TA's in the same place.”*

This sentiment was captured even within research groups. A white third-year Ph.D. student said that his group, “*tends to have everybody kind of off doing their own thing. And while that's fine, it means that if you look at our groups papers there's not a lot of cross pollination.”* Similarly, a Ph.D. student and underrepresented woman of color said that her research group was “*a friendly one”* but that, “*even within in my group, people keep their work separate from each other. We have like separated work that is the same project, but not really collaborating with the same – on the same topic. It's hard to get that collaboration going.”*

The graduate students who did enjoy some kind camaraderie within the department did so in a teaching assistant role. One underrepresented woman of color said that her peer mentor network was, “*The TA's for CS 225*^6^ *for now. They range from like, civil engineering, industrial engineering, also computer science TA's. But it's more like a group of friends, you know?”* An international Master's student said, “*That was one reason why I chose to be a TA. Because, I mean, I figured out that I didn't know a lot of many people in the department after a semester. And then when you're a TA you know a lot of students.”*

When he was seeking a thesis advisor, the same Master's student found mentorship through a teaching assistant, “*I had a TA for one of my courses…and she was in the same area as me. She knew my advisor and she helped me connect with him.”*

#### 4.3.3. Creative sources of mentors

In the written survey, 3 out of 4 undergraduates answered “No” when asked, “Do you have a mentor?” Yet, in addition to the departmental organizations, we found many students seeking creative sources of career and psychosocial support than in 2006. They found support in their peers, family members, friends, and teaching staff.

One Asian undergraduate felt a sense of unease when it came to doing homework with undergraduate men. She said, “*I've had moments where I feel like some male students will ask to work with me. Not because they think I'll be academically like equally successful, or like I'll be very helpful. But just because they want to like…they want to like say that “oh, yea, I worked with her” and like, because I'm a female student.”* She went on to say, “*It feels really weird…do you see me like a peer?”*

We asked whether she had looked into any of the departmental organizations. She said, “*I just feel like I don't really fit in with the people there…I just feel like I don't really connect with them.”* Instead, she had cultivated her own small homework and support network, which included her teaching assistants, engineering learning assistant as well as peers, “*a group of people who will be supportive in that way and not try to boast and like look down on me, you know for not knowing something.”*

In the written survey, a multiracial senior checked the statement, “*I do not need a mentor.”* He elaborated in the interview, “*Not so much that I don't need a mentor, that I think it's more of a student collaboration mentorship. I find a student who has gone through a similar thing and I had talked to them about it. So I have many mentors, kind of one on one, but they're more friends giving friends advice.”*

We asked if he ever followed up with his Faculty Mentor on any of the topics he wanted to discuss. He replied, “*I will always default to another student before I will default to an advisor…”* though he included much of the professional teaching staff and academic office staff in his own mentoring network.

Other sources of mentors were diverse and were often leveraged in times of personal crisis. An white graduate student talked about a family friend, a professional psychologist, that he was able to call in times of struggle. A white undergraduate had a parent and a family friend who both worked on campus; she mentioned them, contrasting these “*fantastic”* sources of mentorship with her discouraging Faculty Mentor interactions.

We are glad that students have support networks, but mentoring should not just be sought as a coping mechanism to deal with the struggles had in the department. Mentoring should also serve as a personal and professional development network that helps all members of the community be engaged in ways that catalyze their successes. Our student participants have expressed wide-ranging negative and harmful mentoring experiences with their tenure-track Faculty Mentors to the degree that they often do not even consider these faculty as part of their mentoring networks and often feel as if their advisors and Faculty Mentors see them as burdens and administrative checkboxes.

One international graduate student pointed out her thesis advisor's mentoring qualities, *he really knows what he's doing. And it's really important to have a mentor that they know their field well. And I couldn't really find a …a good mentor that's like they had a passion about like the next 10 years of their field*.

Aside from graduate students with thesis advisors, we didn't find substantive evidence of research mentorship. One student, a white man, even hesitated talking to his own advisor about career matters. He said, “*Like I, mean I wouldn't bother my advisor whether I should put this on my CV or not.”*

## 5. Positionality

In qualitative research, the stories of both the researcher and the research participants are reflected in the themes that emerge (Kovach, 2009). As such, a statement of our positionality is warranted, given our educational experiences at the university and our professional experiences in the tech industry and the Computer Science Academy. While other human subjects research methodologies may wonder whether our deep connection to the university of inquiry is a too-great source of subjectivity, we view our experiences as an asset. However, with this asset comes a responsibility to set aside time for reflection on our own subjectivity.

Taking inventory of our team, we represent 42 years of experience at the university. From Illinois, we have collectively earned one bachelor's degree, two Master's degrees, and three doctoral degrees. The joys, sleights, and traumas that we experienced on the campus influenced our subsequent professional trajectories. We became senior lecturers, tenured professors, software engineers, and research directors. All the while, we invested energy into the kinds of service that would make the field a better one for underrepresented groups. And, by July 2017, we will all have left the campus.

With these experiences, during the course of our pilot study, we interviewed participants to gather data, but we also saw an opportunity for connection, collegiality and mentorship. As participants shared their own stories, we found moments of celebration in positive shared experiences. For example, when reviewing the variety of student clubs in the department, one student mentioned the Women in Computer Science as a valuable resource. Our own Chambers said, “*I helped found that group…when I was a sophomore. I was one of the first presidents.”* The participant offered up a high-five to celebrate as well as an invitation to rejoin the club's current officers for dinner that evening.

In another moment of celebration, Crenshaw noticed a participant was wearing a particularly unique top. She asked, “*Are you wearing an Apple sweater because you're going to go work there?”* The participant replied, “*I am.”* The participant had already signed an offer letter with the company. The audio erupts into laughter and lots of “*Congratulations!.”*

Not all was celebration. One participant was particularly frustrated with the amount of cheating he was seeing. We commiserated over similar instances and experiences while we were in the department; this allowed us to gather further comments on this culture from the student, including his thoughts on how it began in the department, “*I think there's a lot of students who have been programming for years and consider themselves programmers, but find difficulty doing the assignments and then they think, “well I'm a programmer, one, I could do other important things, and two I don't need this.”*

Another participant who was hungry for more interactions on campus shyly described how he stood out from his peers because he “*played a lot of sports.”* Metcalf told him her own story, “*When I was here, a friend of mine created a Tuesday night volleyball group…And it wasn't just students within this department, but there were engineering students who would join us too. So, connected departments. And then I started a softball group on Sundays. We would all get together and play softball together.”* In a way, sharing her story was a small moment of mentorship for the participant, helping him to see how he might create more opportunities for interaction himself.

Having our own stories to offer in exchange also helped participants feel less alone. We could say with kindness and integrity, “*Yes, we know. We saw it too. That happened to us, too.”* This ability becomes particularly important with sensitive topics like gender discrimination. One participant described a situation where she didn't do anything in response to bad behavior from a student. She said that, “*every time it happens it's so shocking, that I don't feel like “did that even just happen?”*

Metcalf weighed in, “*Because you are shocked at the inappropriateness and you are left speechless about it, right? It's normal for you to feel taken aback and to not really have words in that moment to express a thing.”* She also offered, “*And that is why it's so important to have people who aren't just silent bystanders but who can be allies in those situations. And who are trained to see them and to call people out on those kinds of behaviors. So it's not just the person who's the target of the remark…”*

Some quotes from participants felt eerie, as if words had been taken from our own mouths. At one point, an undergraduate summarized her experiences at Illinois this way, *I hesitate to say this, but I don't encourage women to enter the field right now. It's not a healthy environment. I…I'm not sure if I'm happy that I did it, myself*. There have been multiple moments for all of us when we had to take long breaks from recruiting activities. It is difficult to invite people into a field that can, at times, be so narrow and feel so mean.

It was in these eerie similarities that we realized the importance of our own self-care in this inquiry. Immediately after the pilot interviews in April 2016, we took a month-long break from our weekly conference calls. We described this period as “*Feeling the Feels.”* Chambers wrote a long journal entry during this time, reflecting on how people call her “*one of the lucky ones”* but that it doesn't particularly feel that way. She expresses anger at the times she's been labeled as “*underperforming”* without concrete reason and enumerates the heartbreaking stories of sexual assault she's heard from colleagues, and her own moments where she has been “*left speechless”* by bad behavior. She also reflects on the ways she tries to make her corner of the field a little better. Such reflections resonate with many of the comments shared in our interviewers, and strengthen our feelings of connection to this group.

## 6. Conclusion

This pilot study is the initial step in our follow-up research to understand whether the departmental culture has changed since our original research over 10 years ago, particularly relative to the experiences of women and other underrepresented groups. Our data and discussion above represents only preliminary results from the latest phase in our case study of the University of Illinois' Department of Computer Science. The information gathered in this pilot study has allowed us to update and strengthen our survey and interview instruments.

Our fully-revised data-collection instruments are planned for the Spring 2017 semester where we will recruit participants from the larger departmental population, including faculty, for surveys and follow-up interviews on a variety of topics related to the departmental culture, including: workload; sense of belonging; departmental values; policy awareness, understanding, and effectiveness; sources of mentoring, information, and support; collegiality and respect; classroom experiences; social experiences; research experiences; advising experiences; career goals and development; performance evaluation; communication; perceptions about equity; scientific identity, and more. We will gather more data and conduct additional statistical and thematic analyses that will inform further improvements within the department and contribute to the larger body of research on computing culture. In addition, this will allow us to more fully analyze how the experiences of faculty and students at University of Illinois compare and relate to the broader culture of computing. We will be able to explore a set of recommendations to improve retention and outcomes at Illinois and beyond.

As in other foundational, in-depth case study research (e.g., Margolis and Fisher, 2003), our case study work allows for deeper understanding of the systemic issues affecting computer science retention not only within the case study location, but at similarly situated environments, however defined, beyond it. Such work also inspires new lines of inquiry for a variety of computing environments.

In the meantime, our preliminary findings indicate both improvements and areas of concern. The concrete improvements in undergraduate teaching and the continued success of peer mentoring in the face of increasing enrollment is laudable; in addition, the increased population of women and underrepresented students of color gives hope for the trajectory and future of the department. However, if the department hopes to retain and see success for its growing population of women and underrepresented students of color, several systemic, policy, and cultural issues will need to be addressed.

Descriptions of bias, including acts of violence and differential policy application, exacerbated by policy and resource opacity are particularly concerning. The existence of any one of these areas is likely to contribute to attrition. The incidents of bias against women, students of color, and international students in the classroom, research groups, and online departmental forums left unaddressed by peers, instructors, and faculty sadly align with the expansive body of research on bias, harassment, and discrimination in STEM fields (Hill et al., 2010; Metcalf, 2011; Lincoln et al., 2012; Clancy et al., 2014; Metcalf, 2014; Corbett and Hill, 2015; Moss-Racusin et al., 2015). These indicate the need for bias and harassment training, bystander education, and more effective federal, institutional, and departmental policy implementation and awareness for students, faculty, teaching assistants, and instructors.

The ongoing disconnect between faculty and students, sense of isolation, reliance on peer mentoring networks to cope with negative experiences in the department, perceived inaccessibility of tenure-track faculty, and marginal and negative mentoring experiences also raise retention concerns. Effective mentoring relationships, especially when structured as mosaics, groups, networks, and multiples, positively influence retention, organizational commitment, educational and career development and progression, skill development, knowledge acquisition, and more. Marginal and negative mentoring relationships, however, contribute to stifled careers, attrition, isolation, negative educational and career experiences, and even harmful long-lasting outcomes (Ragins, 1999; Anderson, 2011; Hamlin and Sage, 2011; Baker et al., 2017; Metcalf and Coggin, 2016). The creation of an assigned Faculty Mentor is a positive first step in providing students with the career and psycho-social support needed to be successful; however, the existence of an assigned Faculty Mentor alone cannot yield these outcomes, particularly if the substance of the mentoring relationship is primarily bureaucratic. The quality and content of mentoring relationship matters.

Over the course of a decade, the department has made some substantial progress toward creating a more inclusive culture. While our initial findings are not entirely positive and have been, at times, painful to reflect upon on a personal level, we remain grateful to a departmental leadership willing to do the difficult work of reflection with us—to learn from its struggles and its successes. While some of what we have found thus far is mirrored in the research elsewhere, this unique 10-year look from a cultural vantage point, rather than an individualistic one, provides the possibility of novel interventions in the systemic root causes of inequity and exclusion. As we continue our data collection, we are hopeful that lessons learned will continue to shape positive change in the department for all of its members. In addition, we hope that this multi-phase case study and our future work to expand it to other computing environments will deepen our collective empirical understanding of how systemic, rather than superficial, change unfolds over time and across departmental and institutional contexts.

## Ethics statement

This study was carried out in accordance with the recommendations of the University of Illinois at Urbana-Champaign Institutional Review Board, IRB No. 16507, with written informed consent from all subjects. All subjects gave written informed consent in accordance with the Declaration of Helsinki. The protocol was approved by the IRB.

## Author contributions

This work is a product of all four co-authors. All co-authors have contributed in various degrees to the study's survey instruments, research concept, and analytical methods used.

## Funding

The Department of Computer Science at the University of Illinois at Urbana-Champaign supplied partial funding for our study, supplying travel monies, interview space, a $10 remuneration for each participant, transcription services, and an honorarium for HM.

### Conflict of interest statement

The authors declare that the research was conducted in the absence of any commercial or financial relationships that could be construed as a potential conflict of interest.

## Footnotes

^1^Our materials have received IRB approval from the University of Illinois at Urbana-Champaign, IRB No. 16507. St. Louis University had an Institutional Authorization agreement—or IAA/Reliance Agreement—filed with the University of Illinois, stating they would honor Illinois' IRB decision.

^2^*Source: http://cs.illinois.edu/academics/graduate/phd-program/qualifying-exam*

^3^*Source: https://cs.illinois.edu/about-us/faculty-positions*.

^4^*While the website says there are peer advisors, we found no evidence of a formal peer mentoring system for undergraduate advising*.

^5^*Source:* *http://www.cs.uiuc.edu/academics/undergraduate/undergraduate-advising*.

^6^*An introductory, CS2 course*.
